# Supplementary material for: A mutation in switch I alters the load-dependent kinetics of myosin Va
Source: Nat Commun. 2023 May 30;14:3137. doi: 10.1038/s41467-023-38535-0 (PMC10229639; doi:10.1038/s41467-023-38535-0)
Supplement: Supplementary file 1 — Supplementary Information [file 41467_2023_38535_MOESM1_ESM.pdf]

Supplemental materials

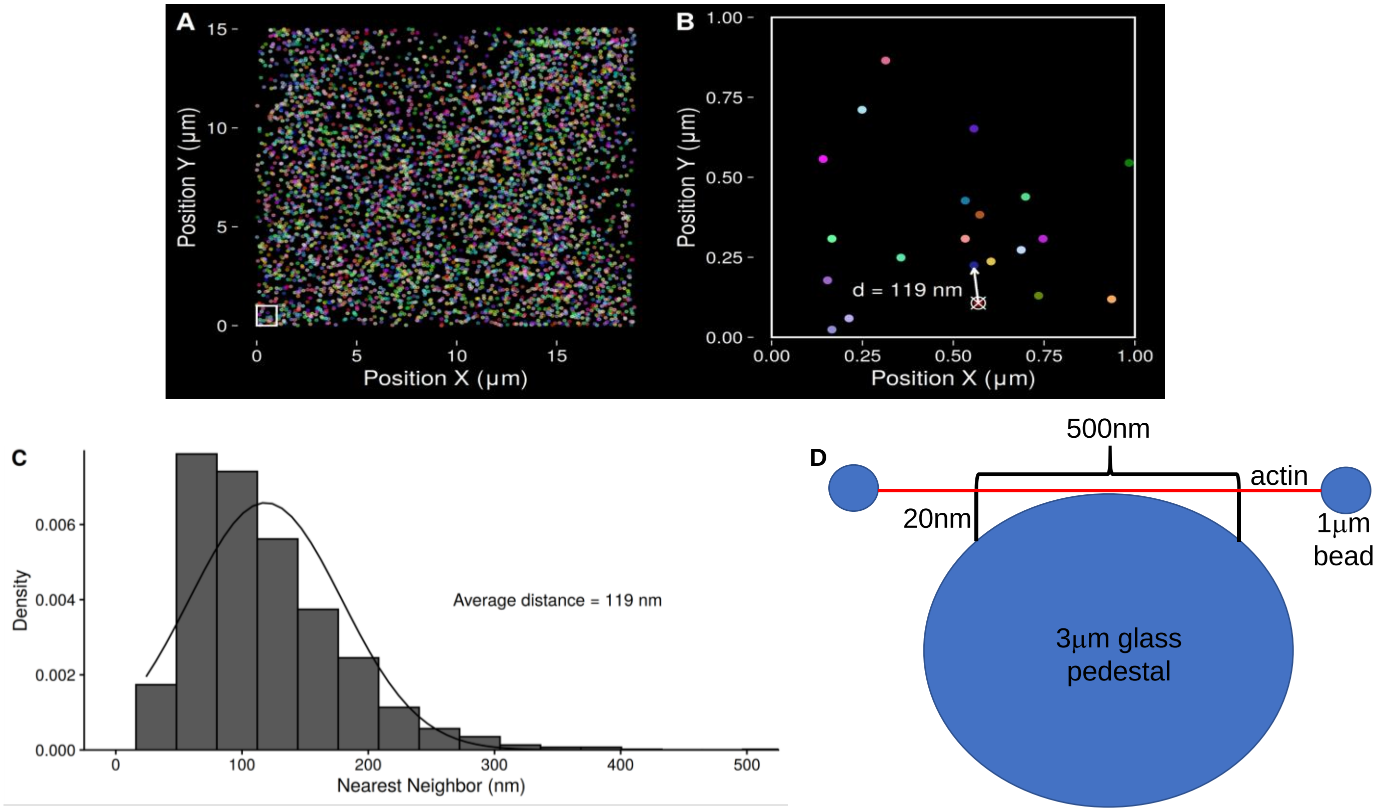

Supplementary Figure 1. STORM estimate of myosin density. A, a representative 2-D image obtained using STORM microscopy with the fluorophores (TRITC) differentially colored to show the density of myosin Va molecules on the surface at the concentration used for the mini-ensembled laser trap assay ($10\mu g \cdot ml^{-1}$). B, an enlargement of the small boxed area in A to show the density in more detail. The distance to the nearest molecule was determined using Nikon Elements® software. Shown is an example of how that distance was calculated. C, the nearest neighbor analysis was determined for the whole field of view (A) and repeated for additional fields and three additional sample chambers to generate the distribution shown in C. The resulting distribution was fit to a Gaussian curve, with a center at 119nm (average distance). This indicates that the space between two myosin molecules was 119nm on average in the mini-ensemble laser trap assay. D, a schematic figure of the three-bead laser trap assay to illustrate the method used to estimate the number of myosin molecules available to interact with the actin filament. Assuming the filament is close enough to contact the 3µm pedestal bead, the length of the actin filament within 20nm (the height of a myosin head) was calculated to be 500nm. Dividing 500nm by the distance between molecules (119nm) suggests that 4.2 molecules on average would be capable of binding to the actin filament.

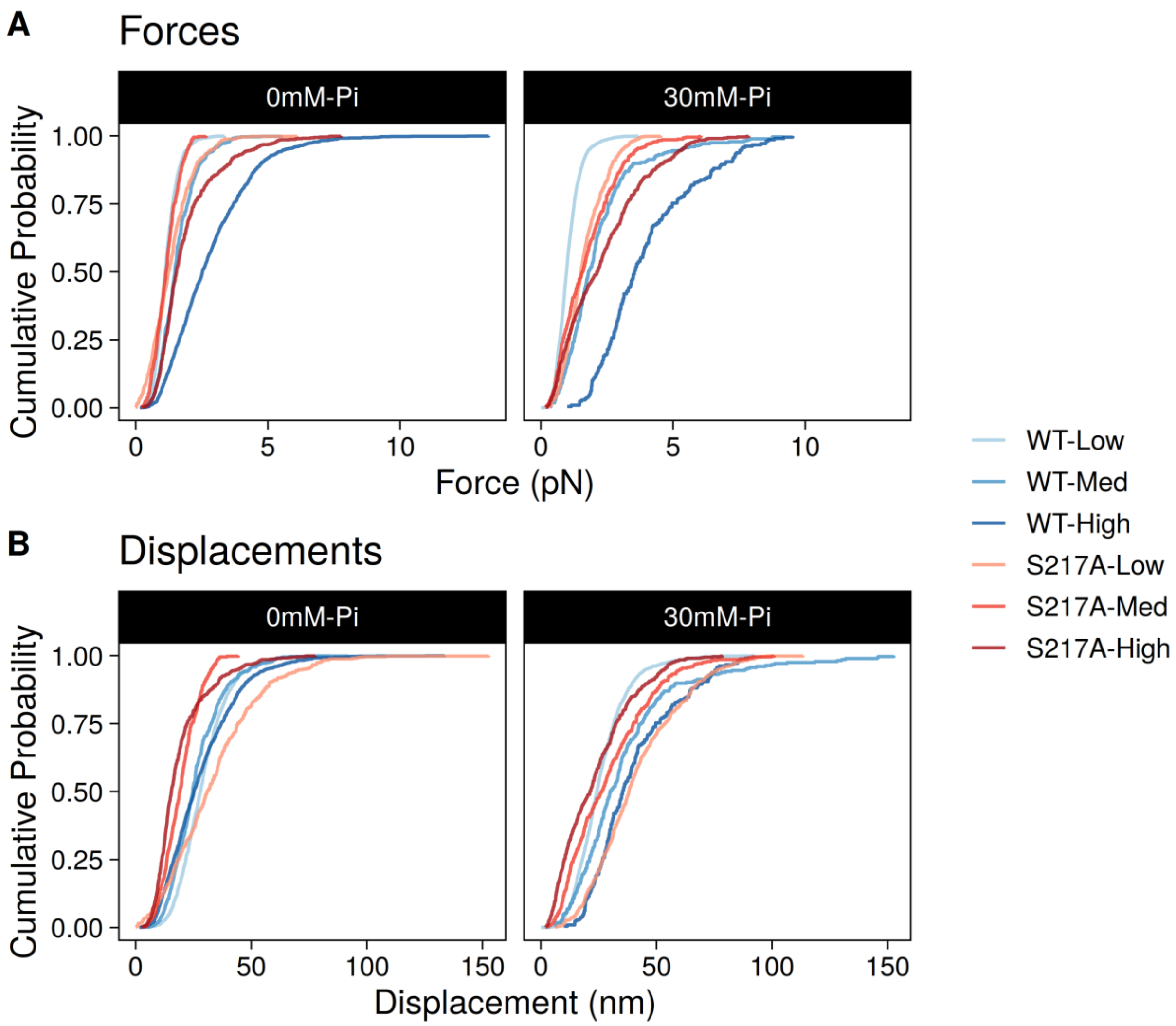

Supplementary Figure 2. Cumulative distributions of force (A) and displacements (B) from the mini-ensemble laser trap assay (see Figure 1 and Methods), at low (0.04 $pN\cdot nm^{-1}$), medium (0.06 $pN\cdot nm^{-1}$) and high (0.10 $pN\cdot nm^{-1}$) laser trap stiffness values. The myosin constructs (WT and S217A) as indicated as well as each level of P_i and trap stiffness are indicated by color according to the legend shown. The average data sets are shown in Figure 3 in the main text.
