# Supplementary material for: A mutation in switch I alters the load-dependent kinetics of myosin Va
Source: Nat Commun. 2023 May 30;14:3137. doi: 10.1038/s41467-023-38535-0 (PMC10229639; doi:10.1038/s41467-023-38535-0)
Supplement: Supplementary file 3 — Reporting Summary [file 41467_2023_38535_MOESM3_ESM.pdf]

nature portfolio

Corresponding author(s): Edward P. Debold

Last updated by author(s): April 27 2023

Reporting Summary

Nature Portfolio wishes to improve the reproducibility of the work that we publish. This form provides structure for consistency and transparency in reporting. For further information on Nature Portfolio policies, see our Editorial Policies and the Editorial Policy Checklist.

Statistics

For all statistical analyses, confirm that the following items are present in the figure legend, table legend, main text, or Methods section.

n/a	Confirmed	
	x	The exact sample size (*n*) for each experimental group/condition, given as a discrete number and unit of measurement
	x	A statement on whether measurements were taken from distinct samples or whether the same sample was measured repeatedly
	x	The statistical test(s) used AND whether they are one- or two-sided<br>*Only common tests should be described solely by name; describe more complex techniques in the Methods section.*
x		A description of all covariates tested
	x	A description of any assumptions or corrections, such as tests of normality and adjustment for multiple comparisons
	x	A full description of the statistical parameters including central tendency (e.g. means) or other basic estimates (e.g. regression coefficient) AND variation (e.g. standard deviation) or associated estimates of uncertainty (e.g. confidence intervals)
	x	For null hypothesis testing, the test statistic (e.g. *F*, *t*, *r*) with confidence intervals, effect sizes, degrees of freedom and *P* value noted<br>*Give P values as exact values whenever suitable.*
x		For Bayesian analysis, information on the choice of priors and Markov chain Monte Carlo settings
x		For hierarchical and complex designs, identification of the appropriate level for tests and full reporting of outcomes
x		Estimates of effect sizes (e.g. Cohen's *d*, Pearson's *r*), indicating how they were calculated

Our web collection on statistics for biologists contains articles on many of the points above.

Software and code

Policy information about availability of computer code

Data collection	The LabView Software is owned by Elliot Scientific Inc. and cannot be shared.
Data analysis	R for data analysis and SPSS for inferential statistical methods. We include the information for the custom R code. And the version for SPSS has<br>been indicated in the Methods

For manuscripts utilizing custom algorithms or software that are central to the research but not yet described in published literature, software must be made available to editors and reviewers. We strongly encourage code deposition in a community repository (e.g. GitHub). See the Nature Portfolio guidelines for submitting code & software for further information.

Data

Policy information about availability of data

All manuscripts must include a data availability statement. This statement should provide the following information, where applicable:

- Accession codes, unique identifiers, or web links for publicly available datasets
- A description of any restrictions on data availability
- For clinical datasets or third party data, please ensure that the statement adheres to our policy

Source data are provided with this paper. The datasets generated and/or analyzed during the current study are available from the corresponding author upon reasonable request. The Source data underlying Fig. 1b are provided as Source Data 1. The Source data underlying Fig. 2a–b are provided as Source Data 2. The

Source data underlying Fig. 3b-f are provided as Source Data 3. The Source data underlying Fig. 4 are provided as Source Data 4.

Research involving human participants, their data, or biological material

Policy information about studies with human participants or human data. See also policy information about sex, gender (identity/presentation), and sexual orientation and race, ethnicity and racism.

Reporting on sex and gender	n.a.
Reporting on race, ethnicity, or other socially relevant groupings	n.a.
Population characteristics	n.a.
Recruitment	n.a.
Ethics oversight	n.a.

Note that full information on the approval of the study protocol must also be provided in the manuscript.

Field-specific reporting

Please select the one below that is the best fit for your research. If you are not sure, read the appropriate sections before making your selection.

[x] Life sciences [ ] Behavioural & social sciences [ ] Ecological, evolutionary & environmental sciences

For a reference copy of the document with all sections, see nature.com/documents/nr-reporting-summary-flat.pdf

Life sciences study design

All studies must disclose on these points even when the disclosure is negative.

Sample size	In a single molecule regime obtaining the number of binding events needed to discern the distribution (e.g. exponential or Poission). This was decided a priori and we determined that the distributions were full by examining the the cumulative distributions. Plots of these distributions and shown for each variable. For further discussion of this idea see https://onlinelibrary.wiley.com/doi/full/10.1002/cyto.a.20000
Data exclusions	none
Replication	Experiments were repeated more five times and were successful every time
Randomization	Samples were separated into WT and mutant protein constructs
Blinding	Investigators were not blinded to the protein used because they had to determine the concentration of each construct before an experiment to ensure the correct number of molecules were tested.

Reporting for specific materials, systems and methods

We require information from authors about some types of materials, experimental systems and methods used in many studies. Here, indicate whether each material, system or method listed is relevant to your study. If you are not sure if a list item applies to your research, read the appropriate section before selecting a response.

Materials & experimental systems

n/a	Involved in the study	
[ ]	[x]	Antibodies
[x]	[ ]	Eukaryotic cell lines
[x]	[ ]	Palaeontology and archaeology
[x]	[ ]	Animals and other organisms
[x]	[ ]	Clinical data
[x]	[ ]	Dual use research of concern
[x]	[ ]	Plants

Methods

n/a	Involved in the study	
[x]	[ ]	ChIP-seq
[x]	[ ]	Flow cytometry
[x]	[ ]	MRI-based neuroimaging

Antibodies

Antibodies used	c-Myc monoclonal antibody (Thermo-Fisher Scientific Inc., catalog # 13-2500) served as a substrate for myosin Va S1 to bind to. The stock 200ug powder was brought up in 100uL of ddH20 and diluted 1:100 before flowing into the flowcell.

Validation

Describe the validation of each primary antibody for the species and application, noting any validation statements on the manufacturer's website, relevant citations, antibody profiles in online databases, or data provided in the manuscript.
